# The biological functions of PRRSV nonstructural protein NSP2 in viral infection and replication and its role in host regulatory mechanisms

**DOI:** 10.3389/fvets.2026.1746188

**Published:** 2026-03-13

**Authors:** Zhenzhen Zheng, Mengsi Zhang, Yue Zhang, Hewei Zhang, Jingqiang Ren

**Affiliations:** 1 Wenzhou Key Laboratory for Virology and Immunology, Institute of Virology, Wenzhou University, Wenzhou, China; 2 College of Food and Drugs, Luoyang Polytechnic, Luoyang, China

**Keywords:** immunomodulation, interaction, NSP2, PRRSV, replication

## Abstract

Porcine reproductive and respiratory syndrome virus (PRRSV) is a single-stranded, positive-sense arterivirus that causes reproductive and respiratory disorders in sows. It is one of the most important pathogens affecting the global swine industry and is widespread worldwide. To date, PRRSV continues to undergo mutation and evolution. NSP2, as the largest non-structural protein generated via proteolytic cleavage of viral polyproteins, has the highest sequence variability among all PRRSV proteins. It cooperates with other non-structural proteins to orchestrate viral replication and modulate host immune responses. This review systematically summarizes the multifaceted functions of NSP2 in the viral life cycle, including its structural and functional characteristics, relationship with viral virulence, mechanisms in viral replication and assembly, role in host immune regulation, and interaction network with both viral and host proteins. These findings provide a theoretical framework for elucidating PRRSV pathogenesis and immune evasion strategies.

## 1 Introduction

Porcine reproductive and respiratory syndrome (PRRS) is a highly contagious and severe disease caused by the PRRS virus (PRRSV). Since the early 1990s, it has spread rapidly worldwide, causing significant damage to the swine breeding industry. In 1992, the World Organization for Animal Health (OIE) officially classified PRRSV as a Category B infectious disease, underscoring its international importance (1).

According to genetic sequence analysis, PRRSV can be classified into two major genotypes: PRRSV-I (European type) and PRRSV-II (American type). The typical representative strain of the European type is the LV strain, whereas the North American type is represented by the VR-2332 strain (2, 3). Among them, PRRSV-II is the prevalent strain in China. As early as 1996, Guo Baoqing and colleagues first successfully isolated the PRRSV from suspected cases and named it strain CH-1a (4). Since then, the virus has spread increasingly widely in China.

In 2004, Gao Zhiqiang isolated the PRRSV HB-2 (sh)/2002P strain and first reported a 12-amino-acid deletion within the NSP2 region (5). As early as 2006, a highly pathogenic porcine reproductive and respiratory syndrome virus (HP-PRRSV) strain swept across many regions of China, resulting in severe morbidity and mortality in pigs. Unlike traditional strains, this variant has a characteristic 30-amino-acid deletion in its NSP2 segment, which distinguishes it from classical strains (6, 7). After 2013, NADC30-like strains with 131 discontinuous amino acid deletions in the NSP2 hypervariable region and NADC34-like strains with a continuous 100-amino acid deletion in the NSP2 region were reported in China (8, 9). These findings indicate that mutations in the NSP2 gene play crucial roles in viral evolution.

Unlike HP-PRRSV, whose NSP2 activates the TLR4/NF-κB pathway to elicit robust inflammation (manifested as acute hyperthermia, pulmonary hemorrhage, and high mortality), the currently prevalent NADC30-like strains harbor a unique 131-amino acid discontinuous deletion (111+1+19) within NSP2. This deletion abrogates efficient binding to the host scaffold protein 14-3-3ζ, resulting in the attenuation of core pro-inflammatory signaling pathways, including MEK1-ERK1/2 and NF-κB, and consequently reduces the production of cytokines such as IL-1β, IL-6, and TNF-α, thereby establishing a low inflammation–high persistence strategy (10, 11). This attenuated immunostimulatory phenotype allows the virus to establish prolonged high-titer infections in tonsils and lymph nodes (with viremia persisting for 3–4 weeks), delays the onset of an effective antibody response, and evades neutralization by antibodies elicited by existing vaccines. Furthermore, through recombination with local strains, it acquires a selective transmission advantage. Collectively, these features enable the virus to maintain a persistent and covert presence within host populations, ultimately manifesting as a clinically mild yet difficult-to-eradicate infection (12).

Therefore, this review systematically examines the biological functions of NSP2, offering insights to inform fundamental PRRSV research and the development of novel prevention and control strategies.

## 2 Structure and function of NSP2

### 2.1 PRRSV and NSP2

**Figure 1:**
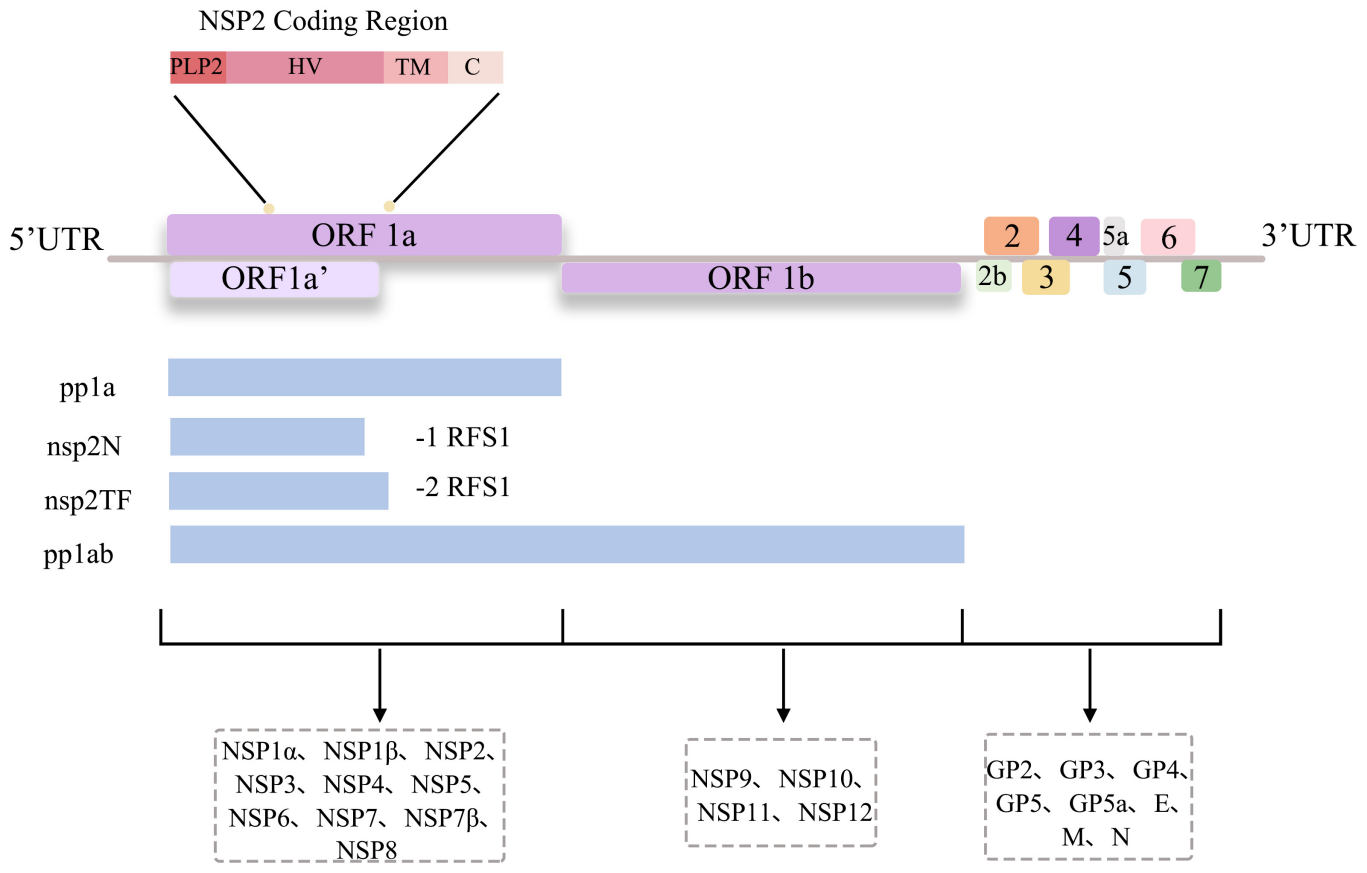
PRRSV genome. The PRRSV genome generates three polyprotein precursors (pp1a, pp1ab, and pp1a-nsp2TF) via −1/−2 programmed ribosomal frameshifts. These precursors are processed by viral proteases to produce 14 non-structural proteins. The structural proteins are translated from sgmRNAs.

PRRSV is a non-segmented, single-stranded, positive-sense RNA virus belonging to the order *Nidovirales*, family *Arteriviridae*, and genus *Arterivirus* (13). Under electron microscopy, PRRSV appears spherical or ellipsoidal with a diameter of 50–55 nm. Its ~15.5 kb positive-sense RNA genome contains a5'-cap, a3'-poly (A) tail, and at least 11 open reading frames (ORFs): ORF1a, ORF1b, ORF2a, ORF2b, ORF3–7, ORF5a, and ORF2TF. ORF1a and ORF1b account for approximately 75% of the genome and encode polyproteins pp1a and pp1ab (through ribosomal frameshifting), which are processed into 14 non-structural proteins (nsps) (14) (Figure 1). ORF2TF is a novel ORF embedded within the NSP2 coding region, which encodes NSP2TF and NSP2N via ribosomal frameshifting (15, 16).

NSP2, the largest nonstructural protein derived from the PRRSV replicase polyprotein (encoded by ORF1a), comprises four structural domains: (1) the N-terminal papain-like cysteine protease 2 (PLP2), (2) the proline-rich hypervariable region (HV), (3) the hydrophobic transmembrane (TM) domain, and (4) the C-terminal conserved domain (17).

Han et al. reported that the PLP2 structural domain and TM structural domain of NSP2 are necessary for PRRSV replication (18). The most important of these domains is the PLP2 structural domain, which has deubiquitinating enzyme (DUB) activity and interferon antagonist function. PLP2 deubiquitinating activity is essential for PRRSV replication.

Through its cis- and trans-cleavage activities (mediated by the catalytic residues C55 and H124), PLP2 autoprocesses from the polyprotein and facilitates the maturation of downstream nonstructural proteins. This proteolytic activity is critical for viral replication complex assembly and function (19). NSP2 generates multiple isoforms (NSP2a–f) during PRRSV replication. These isoforms share an identical N-terminus and form heterodimers on the viral envelope, which are critical for early-stage replication (20, 21). Furthermore, the PLP2 domain mediates cleavage between NSP2 and NSP3, and deletion of both the PLP2 catalytic core (aa 47–180) and its downstream extension (aa 181–323) is required to abolish viral viability (18, 19).

There are a number of nonessential regions (aa324–726) in the highly variable region of NSP2 that are not only able to withstand the modification of a gene deletion or insertion but also able to stably express the exogenously inserted gene fragment [e.g., Myc or GFP (22)]. This property makes NSP2 a valuable tool for recombinant vaccine research, allowing researchers to monitor NSP2 localization and evaluate the effects of insertions on viral replication and virulence. However, these insertions are prone to instability and can be progressively lost during viral replication. For example, Kim et al. reported fluorescence loss in viruses passaged multiple times after EGFP insertion into the NSP2 nonessential region, which poses a new challenge for the development of recombinant viruses as labeled vaccines (22).

Furthermore Phosphorylation of Serine 918 (Ser918) within the hypervariable region of NSP2 functions as a crucial molecular switch that governs viral RNA synthesis. The core mechanism involves the regulation of replication complex stability or viability by the phosphorylation status at this site. Specifically, when Ser918 is phosphorylated (as in the wild-type WT) or in a phospho-mimetic state (S918E mutant), the synthesis of both plus (+) and (–) minus strand genomic RNA remains robust. Concurrently, the relative abundance of subgenomic RNA (e.g., sgRNA2/6/7) is significantly elevated, thereby promoting the efficient expression of downstream viral structural and nonstructural proteins. In contrast, blocking phosphorylation at this site (S918A mutant) leads to a marked reduction in viral replication capacity. This is evidenced by diminished gRNA synthesis and a significant decrease in both the relative abundance and transcription of sgRNAs. Thus, reversible phosphorylation and dephosphorylation of Ser918 serve as a pivotal regulatory node, fine-tuning the efficiency of viral RNA synthesis and ultimately sustaining viral fitness and viability (23).

The TM region of NSP2 is a key region for PRRSV replication. Li et al., in a recent study, revealed the role of NSP2 in virus-mediated host translation shutoff and identified the NSP2 TM region as a key domain responsible for this activity (24). When NSP2 is overexpressed, it significantly inhibits the mammalian target of rapamycin (mTOR) signaling pathway, which, like eIF2α phosphorylation, suppresses host protein translation.

These domains do not function independently, when one loses its function, another domain can compensate for this deficiency or synergistically antagonize the host antiviral response. For example, the HV domain tolerates extensive fragment deletions, likely because other domains compensate for the lost regions, thereby maintaining the virus's fundamental immune evasion capability. The PLP2 domain of NSP2 exhibits DUB activity, removing ubiquitin modifications from signaling molecules to block interferon production. The HV region attenuates immune responses through genetic variation. These mechanisms achieve immune evasion together. Concurrently, the TM domain suppresses host translational shutoff by inhibiting the mTOR signaling pathway, creating favorable conditions for viral replication despite the suppression of overall protein synthesis, and promoting viral escape from host immunity. Additionally, the TM domain and C-terminal tail mediate membrane localization and replication complex assembly, synergizing with PL2's proteolytic activity to establish the viral replication platform, thus ensuring robust viral.

In summary, NSP2 performs distinct functions in different regions during the viral replication cycle. Therefore, studying the structure and function of NSP2 can help further elucidate its role in viral replication and immune evasion and provide a theoretical basis for the subsequent development of novel vaccines.

### 2.2 Impact of NSP2 on PRRSV virulence

Genetic recombination frequently occurs among PRRSV strains. In particular, NSP2 has many deletions and mutations across different PRRSV isolates, resulting in significant polymorphisms. This high degree of variability may be associated with viral adaptation and changes in virulence. Therefore, many researchers have begun to explore the role of amino acid deletions in the hypervariable region of NSP2 in the regulation of PRRSV virulence, aiming to clarify the relationship between amino acid deletions and viral pathogenicity.

Zhou et al. replaced the NSP2 region containing a 30-amino-acid (30-aa) deletion from a highly virulent strain with the corresponding region of an attenuated strain. These findings, combined with animal experimental results, revealed that amino acid variations in NSP2 could not alter viral virulence (25). Researchers subsequently conducted sequence alignment between virulent and attenuated strains and concluded that the determinant factors of virulence may be distributed across the entire viral genome.

On the basis of previous experience, Li et al. analyzed in depth the factors responsible for the highly lethal virulence of PRRSV strains and reported that replacing the region co-coded by NSP9 and NSP10 significantly reduced viral virulence (26). However, the mutant strain HB-1/3.9, which carries NSP9 and NSP10 from HP-PRRSV, does not fully reproduce the characteristics of JXwn 06, suggesting the presence of additional virulence factors.

Recent studies have indicated that the NSP2 gene is an important regulator of virulence. Kong et al. constructed mutant chimeras in the complete coding region of NSP2 in virulent and attenuated strains of HP-PRRSV and reported that NSP2 is a key virulence factor strongly associated with cellular inflammation, immune activation, and the regulation of viral persistence (12).

It is evident that the assertion regarding NSP2 as a virulence determinant remains controversial. We will now attempt to analyze the reasons for this contradiction. Regarding cellular tropism, different strains exhibit varying infectivity toward the same cell type. For example, the vaccine strain HuN4-F112 can be repeatedly passaged in MARC-145 cells but fails to replicate efficiently in porcine alveolar macrophages (PAMs) (27, 28). Notably, the NSP2 residues at positions 893 and 979 in this strain determine infection efficiency during PRRSV infection of PAMs, whereas they do not appear critical *in vivo*. This discrepancy may stem from differences in experimental models and environmental adaptation. Consistent with this, swapping corresponding regions between virulent and attenuated strains fails to alter virulence, a phenomenon likely tied to the genetic background of the strains. Research indicates that replacing the NSP2 of the highly pathogenic HP-PRRSV with that of the low-pathogenic NADC30-like backbone (CHsx1401) requires simultaneous replacement of structural proteins to successfully rescue the virus. The resulting chimeric strain exhibits significantly enhanced virulence and immunopathology, suggesting NSP2 acts as a crucial virulence regulator within specific genetic contexts (12).

Extensive data have confirmed GP5 as the most critical virulence determinant in PRRSV, with amino acid positions 13 and 151 identified as key virulence sites (29). These sites function both independently and synergistically. NSP2 forms complex interaction networks with other PRRSV virulence determinants (GP5, NSP9, NSP10, etc.). For instance, NSP2 directly interacts with GP5 to participate in viral particle assembly and, together with NSP9 and NSP10, forms double-membrane vesicles to promote viral replication.

Therefore, future monitoring of NSP2 genetic variation can be enhanced, which may contribute to a better understanding of PRRSV evolution.

### 2.3 Role of NSP2 in PRRSV assembly and replication

NSP2 is one of the most important viral proteins in the PRRSV life cycle and is involved in multiprotein assembly during the formation of the viral replication complex. Kappes et al. combined the results of immunoelectron microscopy with virus particles purified through multiple rounds of density gradient centrifugation and revealed that isoforms of NSP2 exist both on the surface and in the interior of viral particles (20). These findings indicate that NSP2 can function as a structural protein within viral particles. Recent studies have demonstrated that the full-length NSP2 protein is essential for the formation of double-membrane replication organelles and viral RNA synthesis, and mediates initial viral core assembly by bridging the N protein with envelope proteins (GP2a, GP3, GP4, GP5, M, and E) within the endoplasmic reticulum (ER) and the ER-Golgi intermediate compartment (ERGIC)(30). Conversely, the truncated NSP2TF isoform, while able to bind GP5 and M proteins to prevent their ubiquitin-proteasome degradation and facilitate GP5-M dimerization and viral budding, cannot interact with the N protein and is therefore excluded from nucleocapsid recruitment. Furthermore, NSP2TF localizes to specific compartments of the secretory pathway, which is utilized for viral assembly and release (31).

**Figure 2:**
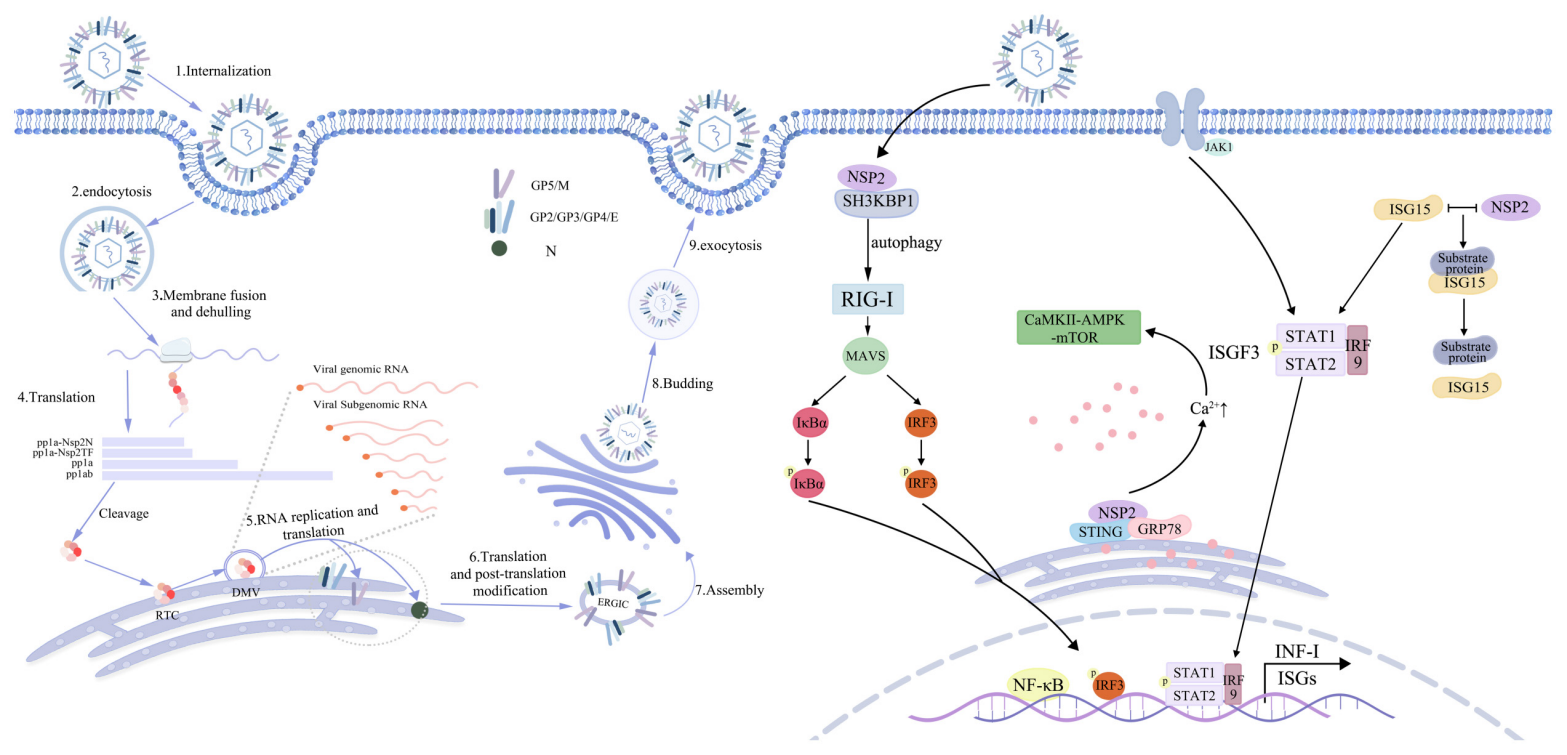
Virus assembly and immunomodulation. The PRRSV infection cycle can be broadly categorized into six stages: attachment, internalization, uncoating, replication, assembly, and release. During this process, the PLP2 domain of the NSP2 protein exhibits deubiquitinating enzyme activity and disrupts the RLR and NF-κB signaling pathways by deubiquitinating key signaling molecules such as RIG-I and IκBα. NSP2 induces autophagy by activating the CaMKII–AMPK–mTOR signaling axis, which in turn leads to the degradation of signaling molecules including MAVS and STAT1/2. Additionally, NSP2 reduces the protein level of ISG15 and dissociates it from its substrates, thereby weakening the host's ISG15-mediated antiviral effects. These mechanisms act synergistically to suppress the expression of type I interferon (IFN-I) and interferon-stimulated genes (ISGs), antagonizing the host's innate immune response.

When infecting eukaryotic cells, all replication activities of positive-stranded RNA viruses take place in the cytoplasm, with their replication and transcription processes occurring within double-membraned vesicles (DMVs). Within these vesicles, viral and host proteins interact collaboratively to assemble the viral replication and transcription complex (RTC), which is responsible for mediating viral replication and transcription. PRRSV nonstructural proteins (NSP2, NSP3, NSP5, NSP9, and NSP10) collaborate to assemble the viral RTC. This complex interacts with the3'-UTR to initiate the transcription of subgenomic mRNAs (sgmRNAs) and the replication of the viral genome (Figure 2). Additionally, mutations in the3'-UTR at positions 117–120 contribute to increased PRRSV replication levels (32). In addition, NSP2 collaborates with NSP3 and NSP5 to form DMVs, thereby creating a robust microenvironment for viral RNA synthesis (33).

Viral proteases target cellular substrates through deubiquitination, thereby interfering with host cell signaling pathways. The NSP2 protein of PRRSV exhibits deubiquitinating enzyme activity and antagonizes interferon signaling, thereby inhibiting interferon production to promote viral replication. NSP2 interferes with type I interferon signaling and promotes PRRSV replication by interacting with STIM1 and subsequently deubiquitinating it, thereby retaining STING in the endoplasmic reticulum (ER) (34). In this process, the deubiquitinase (DUB) activity of the PLP2 domain plays a critical role. Recent proteomic studies have shown that the PRRSV NSP2 protein can interact with the TRIM4 protein. Zhao et al. demonstrated that TRIM4 inhibits PRRSV replication by ubiquitinating and degrading the NSP2 protein (35).

In addition, when the expression of NSP2 was specifically silenced by RNA interference, the replication of PRRSV was inhibited, as manifested by decreased RNA synthesis, reduced protein expression levels, and lower viral titers. Conversely, the level of viral replication was significantly increased when NSP2 was overexpressed, indicating that NSP2 is necessary for the PRRSV replication process (36, 37).

### 2.4 NSP2 and its role in immune regulation

Innate immunity serves as the body's first line of defense against viral invasion. Pattern-recognition receptors (PRRs) detect viral components and trigger immune responses, ultimately leading to viral clearance through the action of interferons and cytokines. Among these, type I interferons are key effectors of the innate immune response, whereas NF-κB is a crucial regulator of both innate and adaptive immunity (38).Previous studies have shown that NSP2 plays a crucial role in inducing the immune evasion mechanisms of PRRSV(Figure 2). PRRSV NSP2 employs multiple pathways to suppress interferon production and subvert interferon signaling, thereby potentiating viral evasion of host immune defenses.

NSP2 is a key protein that suppresses the production of type I interferon. The OTU domain of NSP2 specifically targets the IκBα polyubiquitination pathway, inhibits NF-κB activation, and thereby prevents IκBα degradation. These findings suggest that NSP2 functions as an interferon antagonist to counteract the host's innate immune response (39). RIG-I-like receptors (RLRs) play a key role in the innate immune response by recognizing viral nucleic acids and activating the interferon signaling pathway. Among RLRs, LGP2 inhibits PRRSV replication by interacting with melanoma differentiation-associated gene 5 (MDA5), thereby promoting the activation of interferon regulatory Factor 3 (IRF3) and NF-κB. NSP2 can counteract this antiviral effect by degrading LGP2 via the ubiquitin–proteasome pathway, thereby inhibiting the host immune response and facilitating PRRSV immune evasion (40). Li et al. (41) also revealed a novel mechanism by which NSP2 counteracts the host innate immune response. In their latest study, SH3KBP1 was found to activate the RIG-I signaling pathway by recruiting TRIM25, thereby enhancing the K63-linked polyubiquitination of RIG-I. Using mass spectrometry, researchers identified SH3KBP1 as a novel interacting partner of NSP2. They further demonstrated that the overexpression of SH3KBP1 significantly inhibited PRRSV replication and promoted IFN-β production. Additionally, NSP2 overexpression induced the autophagic degradation of SH3KBP1, thereby attenuating the host innate immune response. The critical interaction region between NSP2 and SH3KBP1 was mapped to the amino acid motif 453PVPAPR458.

In addition, NSP2 can regulate host cell autophagy through multiple mechanisms, thereby influencing the host immune response.NSP2 exerts its function by disrupting the TBK1-IRF3 signaling axis. Mechanistically, it induces TBK1 degradation via chaperone-mediated autophagy. The interaction between NSP2 and the chaperone protein HSPA8 directs TBK1 to lysosomes for LAMP2A-dependent degradation. Consequently, the downstream phosphorylation and nuclear translocation of IRF3 are inhibited, leading to the suppression of type I interferon (e.g., IFN-β) production (42). Zhao et al. found that the PLP2 domain of NSP2 directly interacts with GRASP65 and triggers autophagy by degrading GRASP65, leading to Golgi apparatus disassembly (43). Moreover, Ca^2+^, an important intracellular second messenger, plays a critical role in regulating autophagy. Diao et al. (44) reported that NSP2 induces ER stress by interacting with GRP78 and STIM1, which results in Ca^2+^ release into the cytoplasm and increased intracellular Ca^2+^ concentrations. This, in turn, activates the CaMKII–AMPK–mTOR signaling pathway and promotes autophagy, thereby creating a favorable environment for PRRSV replication. NSP2, as a key effector molecule that induces host translation shutdown, can inhibit the phosphorylation of the mTOR signaling pathway and its downstream signaling molecules (e.g., 4E-BP1). This, in turn, prevents the formation of host translation initiation complexes, thereby enabling immune escape. Thus, targeting NSP2 and its related pathways could help enhance the immune response (45).

NSP2 has also been pinpointed as a significant contributor to inflammatory activation. The NACHT domain of NLRP3 interacts with the transmembrane domain of NSP2. NSP2 facilitates the translocation of NLRP3 to the trans-Golgi network (TGN), facilitating its oligomerization by enhancing the interaction between NLRP3 and IKKβ. This activates the inflammasome and further induces an inflammatory response. The inflammasome is a multiprotein complex integral to the innate immune system, in which the receptor protein NLRP3 serves as a core component. Upon activation, it triggers the release of pro-inflammatory cytokines, such as TNF-α, IL-18, and IL-1β. In PRRSV-infected porcine alveolar macrophage cells, the expression of these pro-inflammatory cytokines is significantly upregulated (46). Du et al. demonstrated that HP-PRRSV NSP2 depends on residues 500-596 and 658-777 within its hypervariable region to activate the COX-2/PGE2 pathway and induce hyperthermia. Through direct interaction with the host protein 14-3-3ζ, NSP2 triggers the MEK1-ERK1/2-C/EBPβ signaling cascade, promoting phosphorylation of ERK1/2 and the transcription factor C/EBPβ. This phosphorylation event subsequently upregulates COX-2 transcription and expression, elevating PGE2 synthesis and ultimately leading to hyperthermia. *In vivo* validation further confirmed that NSP2 mutants lacking these two critical regions are completely devoid of pyrogenic activity (47). These findings further suggest that NSP2 is involved in the innate immune response triggered by the host.

Importantly, autophagy induced by NSP2 can degrade key immune signaling molecules such as TBK1 and SH3KBP1, thereby suppressing interferon production and indirectly weakening the host's antiviral capacity. This immunosuppression creates conditions conducive to the activation of inflammatory responses.

As an immunodominant protein, NSP2 contains six B-cell epitopes and several potential T-cell epitopes. Antibody levels against NSP2, comparable to those against the N protein, can be detected from 1 week to several months post-infection. Moreover, NSP2 effectively induces a humoral immune response (48, 49). The characteristics of PRRSV-induced humoral immunity include the production of abundant non-neutralizing antibodies and delayed emergence of neutralizing antibodies. These non-neutralizing antibodies can specifically recognize PRRSV NSPs; most of these antibodies lack viral neutralizing capacity and may even exacerbate infection by inducing antibody-dependent enhancement (ADE), poses a significant challenge in combating PRRSV infection. Wu et al. showed that anti-NSP serum antibodies were unable to neutralize PRRSV *in vitro*, further demonstrating that antibodies targeting NSPs do not provide protective immunity *in vivo* (50).

The rapid induction of these non-neutralizing antibodies may represent a strategy by which PRRSV evades host immune surveillance, potentially contributing to persistent viral infection and transmission. As previously mentioned, as a strongly immunogenic protein, NSP2 can rapidly induce a large number of non-neutralizing antibodies, thereby delaying the effective immune response against key neutralizing epitopes (such as GP5) on the viral surface. Furthermore, NSP2 itself possesses the ability to interfere with the host's innate immunity. The synergistic effect between these two mechanisms impairs the host's immune capacity, facilitating viral immune escape and the establishment of a persistent infection state. Therefore, for future vaccine development, it is essential to avoid non-essential immunogens and focus more on the development of protective neutralizing antibodies.

## 3 Interactions between NSP2 and viral proteins

### 3.1 Interaction with viral structural proteins

PRRSV encodes eight structural proteins: the major envelope proteins M, N, and GP5; the minor envelope proteins E, GP2, GP3, and GP4; and the newly discovered protein GP5a. Together, these proteins form a complete virion and play essential roles in viral assembly, infection, and replication. For example, GP5 is a highly glycosylated major envelope protein and one of the most important structural proteins capable of inducing neutralizing antibodies. M proteins can form heterodimers with GP5, which is involved in viral invasion. The N protein is the sole component of the nucleocapsid and promotes PRRSV RNA synthesis, thereby influencing viral replication (51–53). Notably, NSP2 can interact with these viral structural proteins to influence viral proliferation.

As early as 2013, Kappes et al. used immunoelectron microscopy to demonstrate the presence of multiple NSP2 isoforms on purified viral particles. These isoforms interact with the N, M, GP2a, and GP5 proteins (20). In addition, two truncated isoforms of NSP2 (NSP2N and NSP2TF) were shown to interact with the M, E, GP3, EGP4, and GP5 proteins. Guo et al. also reported that GP5/M interacts with the NSP2TF protein complex and inhibits its proteasomal degradation by targeting the extracellular pathway (31). This stabilization promotes the formation of GP5-M dimers, which are critical for arterivirus assembly.

In recent studies, researchers revealed the role of NSP2 in viral assembly (30). NSP2 interacts not only with multiple viral envelope proteins but also with the viral nucleocapsid (*N*) protein. More importantly, the N protein can interact with only other envelope proteins (E, M, GP2a, GP3, GP4, and GP5) in the presence of NSP2. Additionally, neither of the two truncated isoforms of NSP2 (NSP2N and NSP2TF) interact with the N protein, suggesting that the structural integrity of NSP2 is essential for viral assembly. These findings provide new insights into the mechanism of PRRSV assembly.

### 3.2 Interaction with non-structural protein

In recent years, the complex interactions among PRRSV nonstructural proteins that coordinate viral replication and transcription during the viral life cycle have become a major focus of research.

Upon infection of host cells, PRRSV induces the remodeling of intracellular membranes to form the DMV, which provides a platform for protein–protein interactions. PRRSV transmembrane proteins are anchored to DMV membranes and recruit other nonstructural proteins through specific interactions, facilitating the assembly of the RTC and promoting viral replication and transcription (54). Among them, NSP2, NSP3, and NSP5 are considered scaffolds of the RTC that induce DMV formation, whereas NSP9 is regarded as the core component of the RTC, with its RNA-dependent RNA polymerase (RdRp) domain being essential for viral replication (55, 56).

Previous studies have shown that NSP2 is indispensable for viral replication and functions as a cofactor of the NSP4 serine protease (57). Li et al. verified that NSP2 and NSP1β co-localize in the perinuclear region of cells, which is a site of viral RNA synthesis (58). Nan et al. mapped the interactions among PRRSV NSPs via yeast two-hybrid and bimolecular fluorescence complementation assays, further confirming a direct interaction between NSP2 and NSP3 (59). Song et al. demonstrated through immunoprecipitation and yeast two-hybrid experiments that NSP2 interacts with NSP1α, NSP1β, NSP9, NSP10, and NSP12(60). These findings collectively support the role of NSP2 in promoting DMV formation and RTC assembly. These studies suggest that NSP2 contributes to the regulation of PRRSV replication through interactions with viral proteins.

## 4 Interactions between NSP2 and host proteins

Virus–host protein interactions play a critical role in the viral replication cycle. PRRSV often enhances its own replication by hijacking host cellular proteins. Given the essential role of NSP2 in viral replication, deciphering its interactions with host proteins is crucial for developing antiviral strategies.

The viral proteins rely on the host's cytoskeletal proteins and molecular chaperones to achieve correct folding, stabilize the viral replication complex, and facilitate intracellular transport and localization. This process further regulates the endoplasmic reticulum stress response and remodels cellular structures to promote viral replication. In the following section, we will elaborate in detail on the molecular mechanisms underlying these interactions.

Heat shock proteins (HSPs) are primarily classified into seven families: HSP100, HSP110, HSP40, HSP60, HSP70, HSP90, and small HSPs. Among these proteins, HSP90AB1 has been shown to interact with NSP2 of PRRSV. Specifically, HSP90AB1 antagonizes the inhibitory effect of NSP2 on NF-κB activity, thereby enhancing NF-κB signaling and ultimately promoting viral replication (61).

NSP2 interacts with other cellular factors. For instance, ATF4 (activating transcription factor 4) is recruited to the viral replication and transcription complex (RTC) through its interaction with NSP2/NSP3, thereby bringing this key unfolded protein response (UPR) transcription factor from the PERK pathway to the sites of RTC localizations (62). This process hijacks host stress-response factors to promote viral RNA synthesis. Additionally, molecular signal-recognition particle 14 (SRP14) interacts with NSP2 and is hijacked to serve the viral RTC, facilitating PRRSV genome synthesis. These findings reveal a host resistance mechanism against PRRSV infection mediated by the IRF8-miR-10a-SRP14 pathway (63).

Vimentin, a type III intermediate filament protein, is widely expressed in cells and plays a role in maintaining the cellular structure and facilitating cell motility. Notably, vimentin is involved in the entry and replication of several viruses (64). It serves as a receptor that mediates PRRSV entry. In a previous study, Song et al. proposed that NSP2 regulates the formation of viral RTCs by indirectly interacting with vimentin, using the N protein as an intermediate (65).

DEAD-box RNA helicase 18 (DDX18) is a member of the DDX family of DEAD-box RNA helicases, which play essential roles in the viral replication cycle. Studies have shown that the knockdown of DDX18 significantly reduces PRRSV replication levels and viral titers, whereas the overexpression of DDX18 increases viral ORF7 copy numbers and viral titers. Furthermore, NSP2 interacts with DDX16 to recruit DDX18 into the RTC, thereby promoting viral replication (66).

In a recent study, Zhu et al. revealed that NSP2 interacts with myeloid-triggered receptor 2 (TREM2) to promote PRRSV replication. TREM2, an anti-inflammatory receptor, modulates host immune responses (67). Knockdown of TREM2 activates the downstream PI3K/AKT and TLR4-NF-κB signaling pathways, leading to early production and release of pro-inflammatory cytokines and type I interferons. Cao et al. showed that 14-3-3ε acts as a pro-virulence factor (68). It is widely involved in various physiological and pathological processes, including innate immunity, the regulation of genome stability, and protein transport. Additionally, 14-3-3ε interacts with the C-terminal domain of NSP2 to induce autophagy, thereby promoting viral infection. In another recent study, Wen et al. demonstrated that NSP2 interacts with SH3 domain-containing kinase-binding protein 1 (SH3KBP1) (69). This interaction blocks the pro-apoptotic activity of SH3KBP1 and reduces PRRSV replication when SH3KBP1 is overexpressed.

**Figure 3:**
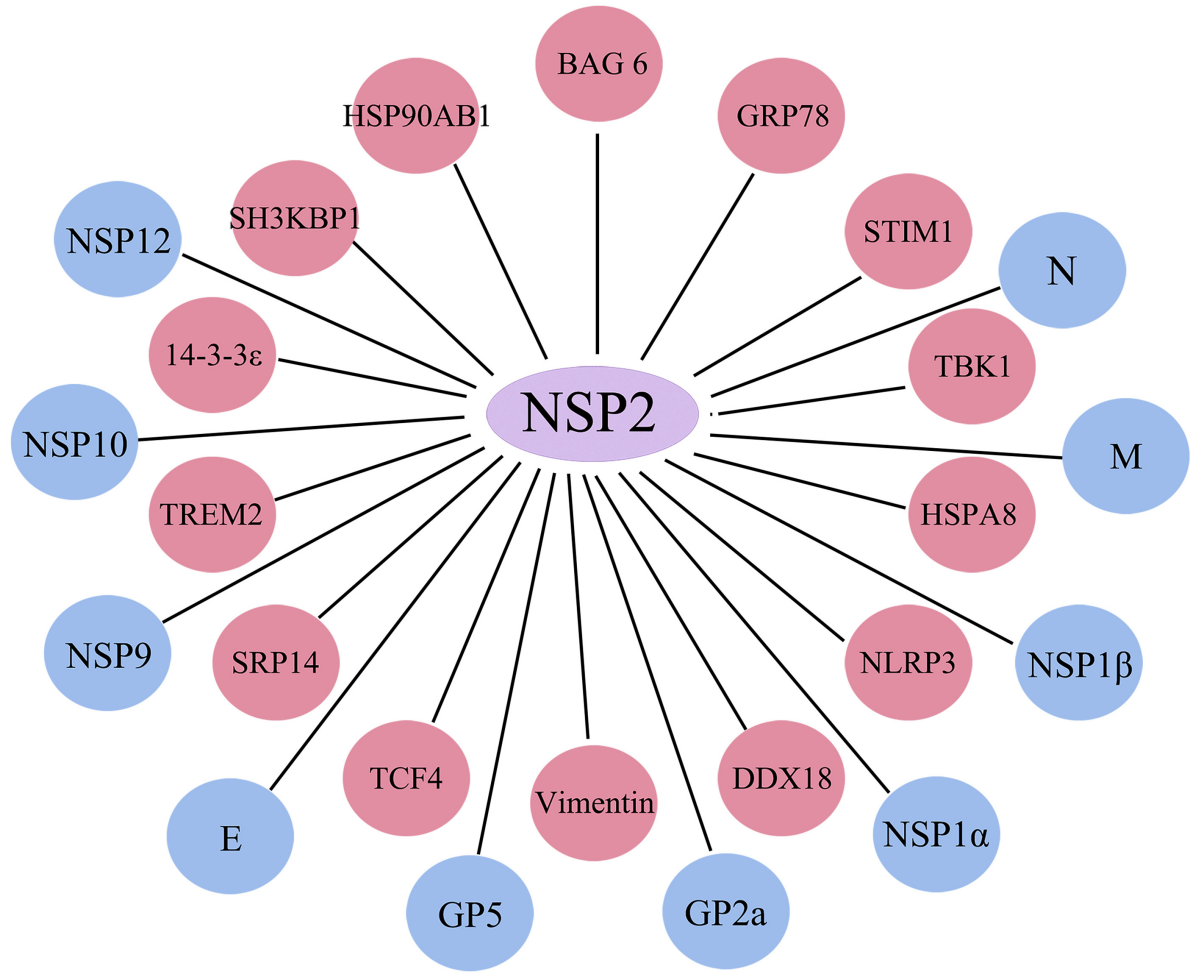
NSP2 protein interaction map. Summary of host proteins and viral proteins interacting with NSP2. Red circles represent host proteins, and blue circles represent viral proteins.

In summary, NSP2 interacts not only with viral proteins but also with a variety of host proteins involved in viral replication and immune regulation, including signaling molecules related to innate immunity, apoptosis, and autophagy (Figure 3).

## 5 Conclusions

Nsp2, the most mutated protein of PRRSV, has a variety of biological functions, including roles in viral replication, assembly, and immunomodulation, but its specific mechanism of action remains unclear. For example, regarding the interaction network between NSP2 and other viral proteins as well as host proteins, although current studies have revealed the existence of complex interactions between them, these interactions have not been further explored, and the role of these interactions in viral infection as well as their dynamic changes are still poorly understood. Furthermore, the three-dimensional structure of full-length NSP2 remains unresolved to this day. Currently, only the crystal structure of the NSP2 OTU domain has been determined. This limitation likely stems from the presence of extensive flexible regions within the highly variable central domain of NSP2, which impede the formation of high-quality crystals suitable for X-ray crystallography or cryo-electron microscopy analysis. Consequently, the three-dimensional structure of NSP2 remains elusive, limiting our understanding of its overall structure-function relationship and synergistic interactions among its distinct domains.

With respect to the involvement of NSP2 in viral particle assembly, recent studies have shown that NSP2 can interact with only coat proteins and membrane proteins; however, the specific mechanism of action has not been clearly investigated. The same problem exists in aspects such as virulence and immunity: the specific regulatory mechanism of NSP2 as a key virulence factor remains unknown; in terms of immunity, many gaps exist, and the specific regulatory mechanism remains to be explored. These problems further limit our understanding of the PRRSV infection process, and the continuous mutation of NSP2 also makes epidemic prevention and control highly difficult.

Therefore, in future research, we can focus on the structure and function of NSP2, further analyze its three-dimensional structure, and understand its function more precisely; in addition, we should strengthen research on the specific molecular mechanisms of these interactions—such as what roles NSP2 plays in them and what functions it performs—which will help us understand the mechanisms of viral infection. Moreover, given the high immunogenicity of NSP2, identifying key sites across its domains can aid in developing targeted inhibitors or vaccines. Among the recombinant vaccines currently developed based on non-essential regions of NSP2, there remains insufficient cross-protection against heterologous strains and an inability to block vertical transmission. These limitations fail to meet existing clinical demands. How to achieve the transition from laboratory research to clinical application remains a critical issue requiring further consideration in the future.

Overall, our understanding of the structure and function of PRRSV NSP2 is limited, and studies focusing on the specific mechanism of action of NSP2 are yet to be performed. Therefore, this paper reviews the structure and function of NSP2 and summarizes its roles in virulence and replication, as well as its interaction network, with the aim of providing insights for basic research on PRRSV.
